# Cajal body formation is regulated by coilin SUMOylation

**DOI:** 10.1242/jcs.263447

**Published:** 2024-12-11

**Authors:** Sara K. Tucker, Douglas M. McLaurin, Michael D. Hebert

**Affiliations:** Department of Cell and Molecular Biology, The University of Mississippi Medical Center, Jackson, MS 39216-4505, USA

**Keywords:** Coilin, SUMOylation, Cajal bodies, Nopp140

## Abstract

Cajal bodies (CBs) are membraneless organelles whose mechanism of formation is still not fully understood. Many proteins contribute to the formation of CBs, including Nopp140 (NOLC1), WRAP53 and coilin. Coilin is modified on multiple different lysine residues by SUMO, the small ubiquitin-like modifier. In addition to its accumulation in CBs, coilin is also found in the nucleoplasm, where its role is still being evaluated. Here, we demonstrate a novel mechanism of CB regulation by examining the interaction changes of coilin when its SUMOylation is disrupted. The impact of global SUMOylation inhibition and targeted disruption of coilin SUMOylation on CB formation was examined. We found that two types of global SUMOylation inhibition and expression of SUMO-deficient coilin mutants increased CB number but decreased CB size. Additionally, we saw via coimmunoprecipitation that a SUMO-deficient coilin mutant has altered interaction with Nopp140. This demonstrates increased mechanistic ties between CB formation and SUMOylation.

## Teaser

**Summary:** The mechanisms driving Cajal body formation are not well understood. We have found that SUMOylation of the Cajal body protein coilin contributes to Cajal body number and size, and influences Cajal body protein interactions.

## INTRODUCTION

Cajal Bodies (CBs) are membraneless organelles found in the nucleus that are involved in the biogenesis of ribonucleoproteins (RNPs) (Kiss, 2004). The presence of CBs can vary based on cell type, developmental stage or transformation status. For example, transformed cell lines (such as HeLa) have a greater percentage of cells with CBs compared to non-transformed cells (such as WI-38) (Hearst et al., 2009; Spector et al., 1992). Enriched within a CB are, in addition to other proteins and non-coding RNAs, the survival of motor neuron protein (SMN, encoded by *SMN1* and *SMN2*), which is mutated in most cases of spinal muscular atrophy; WRAP53, which contributes to telomerase holoenzyme assembly; and coilin, which is canonically known as the CB marker protein (Liu and Dreyfuss, 1996; Mahmoudi et al., 2010; Matera and Frey, 1998; Young et al., 2000). In addition to its localization in CBs, coilin also accumulates in the nucleoplasm. In fact, the majority of coilin is nucleoplasmic in cell types with CBs (Lam et al., 2002). Coilin contains an evolutionarily conserved portion on both the N terminus and C terminus that is separated by an intrinsically disordered region (Bellini and Gall, 1998; Courchaine et al., 2016; Makarov et al., 2013; Tucker et al., 2000). The N terminus of coilin contains a domain most commonly referred to as the ‘self-association’ (SA) domain. This domain is required for coilin–coilin interaction and recruitment into CBs (Hebert and Matera, 2000). Additionally, the C terminus of coilin is also thought to play a role in assembly of CBs (Shpargel et al., 2003). A residue found in the C-terminal domain of coilin, K496, has recently been implicated in contributing to coilin scaffolding function in CBs (Basello et al., 2022). In addition to the association of coilin with itself, it also associates with other proteins such as Nopp140 (also known as NOLC1), a nucleolar phosphoprotein that functions as a molecular link between the nucleolus and CBs (Isaac et al., 1998). Facilitation of this interaction has recently been shown to happen through the coilin N-terminal domain (NTD) (Courchaine et al., 2022).

The condensation and localization of coilin has previously been shown to be dependent upon specific post-translational modifications (PTMs) such as phosphorylation (Hearst et al., 2009; Toyota et al., 2010). Coilin is also modified by SUMO, the small ubiquitin-like modifier (Lett et al., 2023; Xiao et al., 2015), but what effect SUMOylation has on coilin function or CB formation is unknown. Numerous cellular processes are regulated by SUMOylation, including protein stabilization and localization, DNA damage repair, and the immune response (K et al., 2021; Sarangi and Zhao, 2015; Wilkinson and Henley, 2010). SUMOylation is a covalent attachment of a SUMO molecule to a lysine residue of the target protein. The SUMO modification process is similar to ubiquitylation and involves SUMO-specific activating (E1), conjugating (E2) and ligating (E3) enzymes, and the addition of either SUMO-1, SUMO-2, SUMO-3, SUMO-4 or the more recently described SUMO-5 (also known as SUMO1P1) (Liang et al., 2016; Mahajan et al., 1997; Matunis et al., 1996; Wang and Dasso, 2009). SUMOylation also impacts protein–protein interaction through SUMO-interacting motifs (SIMs), which can non-covalently bind to a SUMO conjugated to another protein (Minty et al., 2000). Interestingly, it has previously been shown that overexpressed SUMO-1 localizes to a subset of CBs in stressed neurons (Navascues et al., 2008), and we have shown that coilin promotes protein SUMOylation (Lett et al., 2023).

Similar to CBs, promyelocytic leukemia (PML) bodies are nuclear bodies that contain proteins with various PTMs, including SUMOylation (Hirano and Udagawa, 2022). PML bodies function in a variety of cellular processes such as innate immunity and tumor suppression (Bernardi and Pandolfi, 2007; Bernardi et al., 2008; Salomoni and Pandolfi, 2002), and they contain the PML scaffold protein (Maul et al., 2000). It has been shown that SUMOylation of the PML protein is necessary for formation of PML bodies and proper protein recruitment (Zhong et al., 2000). Additionally, interactions between SIMs and SUMO-conjugated PML contribute to the liquid–liquid phase separation of these nuclear bodies (Van Damme et al., 2010). Since coilin is SUMOylated, it is likely that this modification, as observed for PML bodies, contributes to CB formation and composition, but this remains to be determined.

Here, we conduct a series of experiments designed to examine the impact of SUMOylation on coilin localization and interactions, and on CB formation. Using a combination of SUMO E3 ligase knockdown, SUMO inhibition and coilin mutagenesis experiments, we show that coilin SUMOylation is an important determinate of CB size and number. Since Nopp140 has been shown to be SUMOylated (Xiao et al., 2015), it is likely that interactions between coilin and Nopp140 mediated by SIMs present on both of these proteins also contribute to the regulation of CB size and number. Since coilin is a promoter of SUMOylation (Lett et al., 2023) and other nuclear bodies have been shown to be dependent on SUMOylation for formation, we hypothesize that CB formation is likewise dependent on the SUMOylation of coilin.

## RESULTS

### NSMCE2 knockdown reduces coilin SUMOylation and increases CB number

**Fig. 1.**
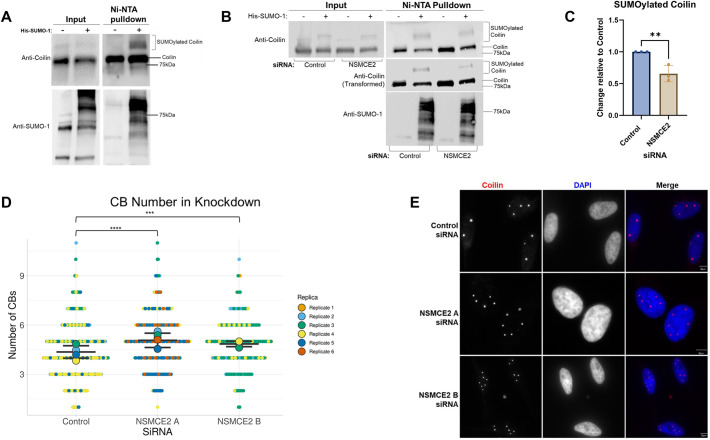
**NSMCE2 KD decreases SUMOylated coilin and increases CB number in HeLa cells.** (A) HeLa cells either remained untransfected or were transfected with His–SUMO-1 for 48 h, as indicated. Ni-NTA pulldown and input (20 μl of total lysate saved and run for inputs) samples were visualized on western blots probed for coilin and SUMO-1. SUMOylated coilin is denoted by a bracket. Blots shown are representative of three experiments. (B) HeLa cells were transfected with control or NSMCE2 A siRNA for 96 h and either untransfected or transfected with His–SUMO-1 (as indicated) at 48 h after KD. After an additional 48 h, protein was collected and subject to Ni-NTA pulldown. Pulldown and input samples (20 μl of total lysate saved and run for inputs) were visualized on western blots probed for coilin and SUMO-1. SUMOylated coilin is denoted by a bracket. A transformed image of the coilin probing is also shown (middle panel) but was not used for quantification. Adjustments to this image were made using the transformation settings on QuantityOne software and applied across the entire image. (C) Quantification of B and replicate experiments, showing the level of SUMOylated coilin normalized to coilin input levels relative to control (which is set to 1) when NSMCE2 is knocked down. Data represent three biological replicates (*N*=3). Bars show the mean, error bars represent s.d. and points represent individual data points. **P<0.01 (two-tailed unpaired Student's *t*-test). (D) SuperPlot of the number of CBs in HeLa cells following control siRNA treatment and two types of NSMCE2 KD for 72 h. ***P<0.001, ****P<0.0001 (two-tailed unpaired Student's *t*-test). CBs were counted by two people who were not aware of the sample identities. Data are from five biological repeats for control siRNA (*n*=389 cells), six biological repeats for NSMCE2 A siRNA (*n*=488 cells) and four biological repeats for NSMCE2 B siRNA (*n*=297 cells). Horizontal line, mean; error bars, s.d.; larger points show the mean for each biological repeat and points represent individual data points. (E) Immunofluorescence of HeLa cells with control siRNA treatment and two types of NSMCE2 KD for 72 h, as indicated. Coilin is shown in red and DAPI (blue) is used to visualize the nucleus. Scale bars: 20 μm. Images are representative of six experiments.

The mechanisms by which the cell regulates the number and size of various nuclear bodies is of great interest. Some insight into the components that impact membraneless organelles, including CBs, has been obtained by an excellent study that used small interfering RNAs (siRNAs) to knock down (KD) expression of more than 1300 human genes and subsequently monitored the morphological changes of nuclear bodies (Berchtold et al., 2018). One protein identified in this previous study as a regulator of CBs is non-structural maintenance of chromosomes (SMC) element 2 (NSMCE2), a component of the SMC5/6 protein complex. Reduction of NSMCE2 results in an increase in CB number (Berchtold et al., 2018). Very interestingly, NSMCE2 is a known SUMO E3 ligase (Potts and Yu, 2005) and has been shown to participate in coilin SUMO-1 modification using a SUMO-activated target trap technique (Salas-Lloret et al., 2023). Taken together, these two studies support the hypothesis that coilin SUMOylation is a regulator of CB formation. To test this hypothesis, we first evaluated whether we could detect endogenous coilin SUMOylation, since our previous study (Lett et al., 2023) examined the SUMOylation of ectopically expressed coilin–GFP. Compared to reactions with untransfected cell lysate, slower migrating endogenous SUMOylated coilin was recovered by nickel-nitrilotriacetic acid (Ni-NTA) beads in lysate expressing His-tagged SUMO-1 (Fig. 1A, upper panel, bracket). Expression of His–SUMO-1 was confirmed via anti-SUMO-1 probing (Fig. 1A, bottom panel).

We next tested the hypothesis that NSMCE2 KD increases CB number by decreasing coilin SUMOylation. Control or NSMCE2 KD lysate was subjected to Ni-NTA pulldown to recover SUMOylated coilin. The amount of SUMOylated coilin was reduced upon NSMCE2 KD compared to levels in the control (Fig. 1B, quantified in Fig. 1C), in agreement with previous results (Salas-Lloret et al., 2023). For this and subsequent quantifications only reactions containing His–SUMO-1 were evaluated. The amount of SUMOylated coilin was normalized to the amount of non-SUMOylated coilin input present in the same samples (Fig. 1B, left panel), relative to that obtained with control siRNA, which was set to a value of 1. The non-specific binding of non-SUMOylated coilin to Ni-NTA beads seen in the Fig. 1B pulldown panel has been reported previously (Lett et al., 2023). Interestingly, many components of small nucleolar RNPs, such as Nop56, Nop58, fibrillarin (FBL) and DKC1, also bind non-specifically to Ni-NTA beads (Ryu et al., 2021). A western blot demonstrating typical NSCME2 KD with two different NSMCE2 siRNAs is shown in Fig. S1A.

After confirming the change in coilin SUMOylation upon NSMCE2 KD, we then examined whether the reduction of NSMCE2 increased CB number in HeLa cells, as previously reported (Berchtold et al., 2018). For this assay, cells were treated with control siRNA or one of two different NSMCE2 siRNAs, followed by immunofluorescence analysis with coilin antibodies to detect CBs. We observed a significant increase in CB number in HeLa cells when NSMCE2 was reduced with either NSMCE2 A siRNA (5.1 CBs per cell) or NSMCE2 B siRNA (4.8 CBs per cell), as compared to CB numbers in cells treated with control siRNA (4.4 CBs per cell). A SuperPlot containing all the data for each siRNA and replicate is shown in Fig. 1D. Representative images showing CBs for each siRNA treatment are shown in Fig. 1E. Coilin foci observed upon NSMCE2 KD were verified to be CBs by staining for SMN (Fig. S1B). Although previous work has shown that NSMCE2 KD in HeLa cells increases the percentage of cells in G2-M transition (Behlke-Steinert et al., 2009), we did not observe any obvious change in proliferation in our KD conditions. Interestingly, evaluation of HeLa CB number throughout the cell cycle has shown that CBs are smaller and greater in number in mid- to late-G1 phase and then become larger and fewer in number in S and G2 phases (Andrade et al., 1993). Since we observed that NSMCE2 KD results in more and smaller CBs, this phenotype is not what one would expect if NSMCE2 KD increased the percentage of cells arrested at the G2-M transition. To confirm this, we performed flow cytometry to assess the changes in cell cycle distribution between control siRNA-treated cells and NSMCE2 siRNA-treated cells. Similar to previously published studies, we found significantly more cells in G2 following NSMCE2 KD, confirming G2-M arrest (Fig. S1C,D). These findings mechanistically connect two previous studies (Berchtold et al., 2018; Salas-Lloret et al., 2023) and demonstrate that NSMCE2 regulation of coilin SUMOylation correlates with CB number and is not the result of a block in G1 phase.

### The SUMOylation inhibitor TAK-981 reduces coilin SUMOylation and increases CB number

**Fig. 2.**
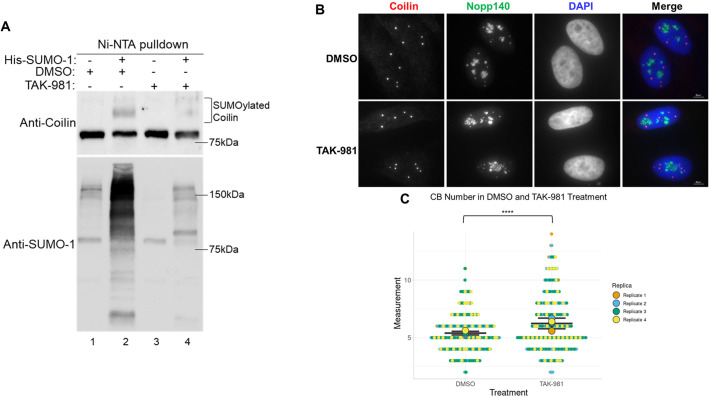
**The SUMOylation inhibitor TAK-981 decreases SUMOylated coilin and increases CB number in HeLa cells.** (A) HeLa cells were transfected either with or without His–SUMO-1 for 48 h. Cells were treated with either DMSO or TAK-981 (0.1 µM) for 24 h post-transfection, as indicated. Protein was subject to Ni-NTA pulldown. Pulldown was visualized on a western blot probed with anti-coilin and anti-SUMO-1 antibodies. SUMOylated coilin is denoted by a bracket. Blots shown are representative of three experiments. (B) Immunofluorescence of HeLa cells treated with either DMSO or TAK-981 (0.1 µM) for 24 h. Coilin is shown in red and Nopp140 is shown in green. DAPI (blue) is used to visualize the nucleus. Scale bars: 20 μm. (C) SuperPlot showing number of CBs in HeLa cells treated with either DMSO or TAK-981. A total of 322 cells were counted from four biological replicates for both DMSO and TAK-981 treatments. Horizontal line, mean; error bars, s.d.; larger points show the mean for each biological repeat and points represent individual data points. ****P<0.0001 (two-tailed unpaired Student's *t*-test).

To further demonstrate the role of SUMOylation in CB formation, we monitored coilin SUMOylation and CB number in HeLa cells treated with TAK-981. TAK-981 inhibits the SUMO activating enzyme (E1) and thereby globally inhibits SUMOylation (Langston et al., 2021). We verified that TAK-981 inhibits SUMOylation by treating HeLa cells with different amounts of TAK-981 and monitoring the global level of SUMO-2 and/or SUMO-3 (SUMO-2/3)-modified proteins (Fig. S2A). Similar to NSMCE2 KD, TAK-981 has previously been shown to affect the cell cycle, causing arrest at the G2-M transition (Kumar et al., 2022). Again, to confirm this we performed flow cytometry to assess the changes in cell cycle distribution between DMSO-treated cells and TAK-981-treated cells, and found significantly more cells in G2 and significantly fewer cells in G1 with TAK-981 treatment (Fig. S2B,C). To evaluate whether TAK-981 treatment decreases coilin SUMOylation, untransfected HeLa cells and HeLa cells transfected with His–SUMO-1 were treated 24 h later with vehicle (DMSO) or 0.1 µM TAK-981. After 24 h treatment, cell lysates were subjected to Ni-NTA pulldown. The amount of slower migrating SUMOylated coilin was reduced by TAK-981 treatment, compared to that obtained with vehicle treatment (Fig. 2A, upper panel, compare lane 4 to lane 2). Probing the same blot with anti-SUMO-1 showed that global recovery of SUMO-1-conjugated proteins by Ni-NTA beads was reduced upon TAK-981 treatment, compared to the recovery from DMSO-treated samples (Fig. 2A, lower panel, compare lane 4 to lane 2).

To evaluate whether CB number is altered by TAK-981, cells were treated with DMSO or 0.1 µM TAK-981 followed by immunofluorescence to detect coilin and Nopp140. Nopp140 localizes to CBs and the nucleolus, and interactions between the N terminus of coilin with Nopp140 are important for CB formation (Isaac et al., 1998; Courchaine et al., 2022). By staining for Nopp140 and coilin, we could assess whether the inhibition of SUMOylation by TAK-981 alters the localization of Nopp140 in CBs. With the conditions used here, no obvious change in Nopp140 localization was detected in TAK-981-treated cells compared to DMSO-treated cells (Fig. 2B). We did, however, observe a statistically significant increase in CB number in cells treated with TAK-981 (6.2 CBs per cell) compared to DMSO (5.35 CBs per cell) (Fig. 2C). Further analysis of these data revealed that whereas only 10% of DMSO-treated cells contained counts of eight or more CBs (32 out of 322 cells), nearly three times that amount (27%) was found upon TAK-981 treatment, with 88 out of 322 cells having eight or more CBs. These findings are not due to a G1 block by TAK-981 since cell cycle analysis showed that cells are enriched in G2-M transition by this treatment (Fig. S2B,C), and CBs are larger and fewer in number in S and G2 phases (Andrade et al., 1993). Reduction of coilin SUMOylation, as a consequence of NSMCE2 KD or inhibition of SUMOylation by TAK-981, therefore correlates with an increase in CB number.

### Expression of SUMO-deficient coilin mutants increases CB number in HeLa cells

**Fig. 3.**
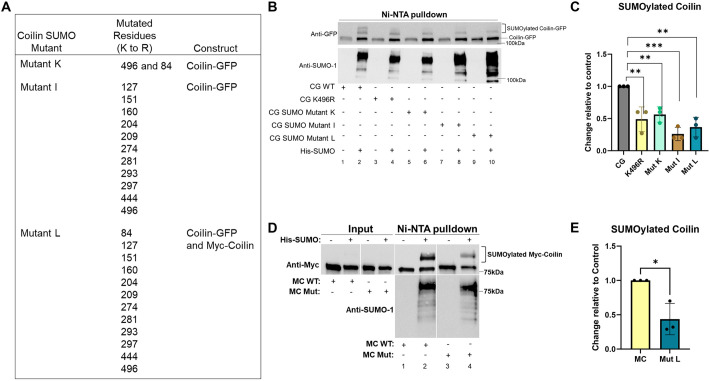
**Coilin SUMO mutants show decreased SUMOylation.** (A) The mutants constructed, with mutated lysine residues indicated. (B) HeLa cells were transfected with WT or mutant coilin–GFP (CG), with or without His–SUMO-1 for 24 h, as indicated. Protein was collected and subject to Ni-NTA pulldown. Pulldown was visualized on western blots probed with anti-GFP and anti-SUMO-1 antibodies. SUMOylated coilin is denoted by a bracket. (C) Quantification of B and repeat experiments showing SUMOylated coilin in the presence of the different mutants. Data represents three biological replicates for WT coilin–GFP (CG), coilin–GFP K496R, coilin–GFP mutant K (Mut K), coilin–GFP mutant I (Mut I) and coilin–GFP mutant L (Mut L). Bars show the mean, error bars represent s.d. and points represent individual data points. **P<0.01, ***P<0.001 (two-tailed unpaired Student's *t*-test). (D) HeLa cells were transfected with WT myc–coilin (MC WT) or myc–coilin mutant L (MC Mut) with or without His–SUMO-1 transfection for 24 h, as indicated. Protein was collected and subject to Ni-NTA pulldown. Pulldown and input samples (20 μl of total lysate was run as input) were visualized on western blots probed for myc and SUMO-1. SUMOylated coilin is denoted by a bracket. (E) Quantification of D and repeat experiments showing SUMOylated coilin changes for WT myc–coilin (MC) and myc–coilin mutant L (Mut L). Data represents three biological replicates. Bars show the mean, error bars represent s.d. and points represent individual data points. *P<0.05 (two-tailed unpaired Student's *t*-test).

We next decided to hinder coilin SUMOylation by creating lysine-to-arginine mutations of coilin lysine residues that are reported in UniProt as being SUMOylated (UniProt:P38432; Apweiler et al., 2023), or are predicted to be SUMOylated by JASSA (Beauclair et al., 2015) and Abcepta (available at https://www.abcepta.com/sumoplot) SUMO prediction software. Mutant coilin constructs contained a C-terminal GFP tag (coilin–GFP) or an N-terminal myc tag (myc–coilin). An N-terminal GFP tag coilin construct (GFP–coilin) was not used for mutagenesis because we have previously shown that GFP–coilin is not efficiently SUMOylated compared to coilin–GFP or myc–coilin (Lett et al., 2023) (Fig. S2D). We also note that GFP–coilin and coilin–GFP localize differently (Hebert and Matera, 2000). Specifically, GFP–coilin tends to have a greater nucleoplasmic signal compared to coilin–GFP, which is mostly enriched in numerous foci with little nucleoplasmic signal. Although these coilin–GFP foci can be many in number, they colocalize with Sm proteins and fibrillarin, which is consistent with coilin–GFP foci being CBs (Hebert and Matera, 2000). Additionally, significant overexpression of GFP–coilin abolishes CBs, whereas overexpression of coilin–GFP can generate multiple foci that can sometimes merge to form large blobs almost the size of nucleoli (Hebert and Matera, 2000). However, low and medium levels of coilin–GFP generate foci that contain CB epitopes such as Sm proteins, fibrillarin, Nopp140 and SMN (Hebert and Matera, 2000), and many labs routinely use coilin–GFP in their analysis of CBs (for example, Courchaine et al., 2022). Since GFP–coilin shows less relative SUMOylation compared to coilin–GFP and endogenous coilin (Fig. 1A; Fig. S2D; Lett et al., 2023), and GFP–coilin does not form many CBs in a primary cell line (Hearst et al., 2009; Toyota et al., 2010), we utilized the coilin–GFP construct for our analysis and verified that foci formed were CBs by detection of SMN. Cells overexpressing the coilin constructs were not analyzed. The lysine residues mutated were 84, 127, 151, 160, 204, 209, 274, 281, 293, 297, 444 and 496. The majority of these known or predicted SUMOylated lysine residues are in the central intrinsically disordered region of coilin. K496 has been shown to be SUMOylated (Xiao et al., 2015) and impairs CB formation when mutated to glutamic acid (Basello et al., 2022). Importantly, it has previously been shown by mass proteomic analysis that residue 496 is also ubiquitylated, which might be a modification that offers an alternative explanation for any changes seen with constructs that include mutations of this residue (Basello et al., 2022). A fully mutated coilin construct, indicated as mutant L, therefore contains 12 lysine residues converted to arginine (Fig. 3A).

**Fig. 4.**
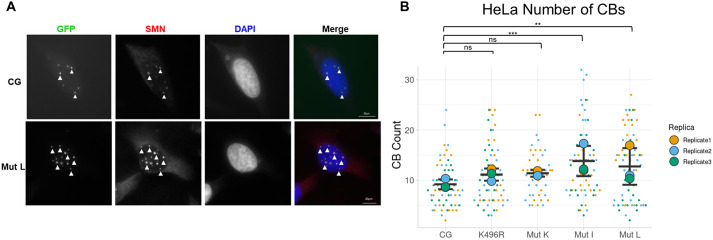
**Coilin SUMO mutants show altered CB phenotypes.** (A) Immunofluorescence of HeLa cells transfected with WT coilin–GFP (CG) or coilin–GFP mutant L (Mut L) for 24 h and stained for SMN. WT and mutant coilin–GFP are shown in green, SMN is shown in red. DAPI (blue) is used to visualize the nucleus. Arrowheads indicate CBs. Scale bars: 20 μm. (B) SuperPlot of the number of CBs in HeLa cells transfected with the different coilin–GFP SUMO mutants (CG, WT coilin–GFP; Mut I, mutant I; Mut K, mutant K; Mut L, mutant L). At least 60 cells from three biological replicates were scored for all constructs except mutant K, for which 44 cells from two biological replicates were scored. Horizontal line, mean; error bars, s.d.; larger points show the mean for each biological repeat and points represent individual data points. **P<0.01; ***P<0.001; ns, not significant (two-tailed unpaired Student's *t*-test).

To monitor the degree that given mutations decreased coilin SUMOylation, coilin–GFP wild-type (WT) or mutant plasmid constructs were transfected into HeLa cells alone or in combination with His–SUMO-1 plasmid, and lysate was subjected to Ni-NTA pulldown. Normalized to the amount of coilin–GFP (or mutant) in the input lanes (Fig. S2E), the amount of SUMOylated WT coilin–GFP was greater than that of SUMOylated coilin–GFP K496R, coilin–GFP mutant K (K84R and K496R), coilin–GFP mutant I or coilin–GFP mutant L (Fig. 3B, quantified in Fig. 3C with WT coilin–GFP set to a value of 1). Interestingly, the amount of SUMOylated coilin–GFP mutant I and coilin–GFP mutant L was significantly lower than the amount of SUMOylated coilin–GFP K496R (*P*=0.0002 and *P*=0.002, respectively). Furthermore, the amount of SUMOylated coilin–GFP mutant I was slightly but significantly less than that of SUMOylated coilin–GFP mutant K (*P*=0.003), indicating that lysine residues in addition to K84 and K496 are SUMOylated, which is in agreement with proteomic studies (Xiao et al., 2015). We also constructed mutant L in a myc–coilin background and found significantly less SUMOylated myc–coilin mutant L compared to levels of SUMOylated WT myc–coilin (Fig. 3D, quantified in Fig. 3E). Again, the amount of SUMOylated coilin was normalized to the amount of non-SUMOylated coilin input present in the same samples (Fig. 3D, left panel) and was expressed relative to that obtained with WT myc–coilin transfection, which was set to a value of 1. We did not see any large changes in expression levels between the various coilin mutants utilized. Next, we asked whether these SUMO-deficient coilin mutants altered CB number. For this analysis, HeLa cells were transfected with WT or mutant coilin–GFP, and CBs were evaluated. Cells transfected with coilin–GFP constructs with progressively more mutated lysines, such as mutant L, had more CBs compared to those transfected with WT coilin–GFP (Fig. 4A). Like foci formed by WT coilin–GFP, CBs formed by coilin–GFP mutant L colocalized with SMN (Fig. 4A). Quantification of CB number indeed showed that cells transfected with coilin–GFP mutant I (13.8 CBs per cell) or coilin–GFP mutant L (12.7 CBs per cell) had significantly more CBs compared to cells transfected with WT coilin–GFP (9.7 CBs per cell) (Fig. 4B).

### Differential interaction of Nopp140 with coilin SUMO-deficient mutants

**Fig. 5.**
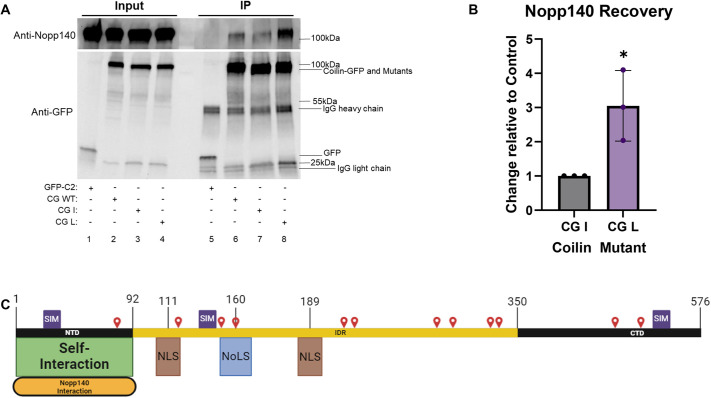
**Coilin SUMO mutants have altered interaction with Nopp140.** (A) HeLa cells were transfected with either GFP-C2 empty vector, WT coilin–GFP (CG WT), coilin–GFP mutant I (CG I) or coilin–GFP mutant L (CG L) for 24 h, as indicated. Lysate was subject to immunoprecipitation (IP) with anti-GFP antibody. IP and input (20 μl of total lysate was run as input) samples were run on SDS-PAGE followed by western transfer and probing for Nopp140 (upper panel) or GFP (lower panel). (B) Quantification of A and repeat experiments, showing Nopp140 signal normalized to recovered coilin–GFP mutant I or coilin–GFP mutant L, with the mutant I ratio set to 1. Bars show the mean, error bars represent s.d. and points represent individual data points. *P<0.05 (two-tailed unpaired Student's *t*-test). (C) Schematic of coilin, showing the NTD, C-terminal domain (CTD) and intrinsically disordered region (IDR), with lysine residues that were mutated to arginine residues denoted as red pins and amino acid residue numbers marked. Self-interaction region, Nopp140 interaction region, nuclear localization signals (NLS) and a nucleolar localization signal (NoLS) are shown, as are SIMs.

As previously mentioned, coilin condensation has also been linked to the association of Nopp140 with the coilin NTD (Courchaine et al., 2022). We next wanted to see whether coilin mutants that alter CB number also alter interaction with Nopp140. For this analysis, lysate from HeLa cells transfected with empty GFP vector (GFP-C2), WT coilin–GFP, coilin–GFP mutant I or coilin–GFP mutant L was subject to immunoprecipitation with anti-GFP antibody followed by detection of Nopp140. As expected, Nopp140 was not recovered in reactions using lysate from cells transfected with empty GFP vector (Fig. 5A, upper panel, lane 5). However, Nopp140 was coimmunoprecipitated by WT coilin–GFP, coilin–GFP mutant I and coilin–GFP mutant L (Fig. 5A, upper panel). The same blot was also probed with anti-GFP, and approximately equal amounts of coilin–GFP or mutants were immunoprecipitated (Fig. 5A, lower panel). We consistently observed that more Nopp140 was recovered, relative to the amount of immunoprecipitated coilin–GFP or mutant construct, in reactions with coilin–GFP mutant L compared to that obtained with coilin–GFP mutant I. Quantification showed that significantly more Nopp140 was recovered by coilin–GFP mutant L relative to that found for coilin–GFP mutant I, which was set to a value of 1 (Fig. 5B). There is only one difference between the mutant I and mutant L constructs: K84 is mutated to R in mutant L (Fig. 3A). K84 is not listed in UniProt as a residue known to be SUMOylated. However, this residue is predicted to have the highest probability of being SUMOylated amongst all coilin lysines by both the JASSA and Abcepta SUMO prediction tools. Very interestingly, K84 resides in the NTD of coilin, which interacts with Nopp140 (Fig. 5C; Courchaine et al., 2022). Since coilin–GFP mutant L, which contains K84R, recovered more Nopp140 compared to coilin–GFP mutant I, these findings are consistent with SUMOylation of K84 being a negative regulator of the coilin–Nopp140 interaction.

### SUMO-deficient coilin mutants increase CB number and decrease CB size in the human foreskin fibroblast primary cell line

**Fig. 6.**
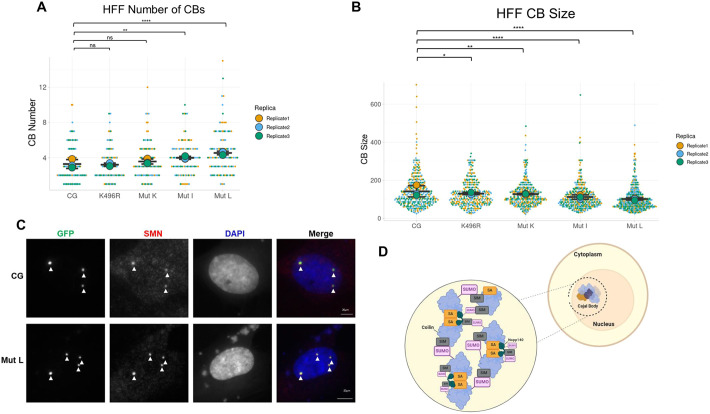
**Coilin SUMO mutants display dysregulated CB formation in HFF cells.** (A) SuperPlot of number of CBs in HFF cells when transfected with different mutants for 24 h. Three biological repeats were scored. *n*=125 cells scored for WT coilin–GFP (CG), *n*=115 cells scored for coilin–GFP K496R (K496R), *n*=105 cells scored for coilin–GFP mutant K (Mut K), *n*=106 cells scored for coilin–GFP mutant I (Mut I) and *n*=117 cells scored for coilin–GFP mutant L (Mut L). ****P<0.0001; **P<0.01; ns, not significant (two-tailed unpaired Student's *t*-test). (B) SuperPlot of CB size (pixels) in HFF cells when transfected with different mutants for 24 h. CB size was measured from three biological repeats. *n*=405 CBs measured for WT coilin–GFP, *n*=369 CBs measured for coilin–GFP K496R, *n*=379 CBs measured for coilin–GFP mutant K, *n*=419 CBs measured for coilin–GFP mutant I and *n*=533 CBs measured for coilin–GFP mut L. *P<0.05, **P<0.01, ****P<0.0001 (two-tailed unpaired Student's *t*-test). In A and B, horizontal lines mark the mean, error bars represent s.d., larger points show the mean for each biological repeat and points represent individual data points. (C) Representative immunofluorescence images of HFF cells transfected with WT coilin–GFP (CG) or coilin–GFP mutant L (Mut L) for 24 h and stained for SMN. GFP signal is shown in green. SMN staining is in red. DAPI (blue) is used to visualize the nucleus. Arrowheads indicate some of the CBs. Scale bars: 20 μm. (D) Proposed schematic of the involvement of coilin and Nopp140 SUMOylation in CB formation. SA, self-association domain.

We next wanted to evaluate the CB formation potential of SUMO-deficient coilin mutants in a cellular background with few CBs. Unlike transformed cell lines like HeLa that have numerous CBs in most cells, primary non-transformed cell lines like WI-38 have relatively few cells with CBs, and those cells with CBs typically only have one or two (Spector et al., 1992). For our analysis, we utilized the non-transformed human foreskin fibroblast (HFF) cell line, in which ∼10% of cells have CBs and CB-containing cells typically only have a single CB (Fig. S3A). Transfection of HFF cells with a GFP–coilin construct results in nucleoplasmic GFP–coilin signal in most cells (Fig. S3B), with some cells having one CB. Consequently, we utilized coilin–GFP constructs for these studies as we had done for HeLa cells. HFF cells were transfected with WT coilin–GFP or mutants thereof for 24 h, and the GFP signal was evaluated. CB number per cell was scored for each construct from three biological repeats, revealing that cells transfected with coilin–GFP mutant I and coilin–GFP mutant L had significantly more CBs per cell (4.0 and 4.5, respectively) than cells transfected with WT coilin–GFP (3.3 CBs per cell) (Fig. 6A). In the process of counting CBs, we observed that CBs seemed to be smaller in cells expressing coilin–GFP mutants I and L compared to those in cells expressing WT coilin–GFP. To quantify this difference, CB size was measured in three biological repeats for each construct. For all mutant constructs, cells expressing mutant coilin–GFP showed significantly smaller CBs, quantified in pixels, compared to those in cells expressing WT coilin–GFP. Specifically, average CB size was 146 pixels for WT coilin–GFP, 131 pixels for coilin–GFP K496R, 126 pixels for coilin–GFP mutant K, 113 pixels for coilin–GFP mutant I and 102 pixels for coilin–GFP mutant L (Fig. 6B). Additionally, CB size was found to be significantly smaller for coilin–GFP mutant L than it was for coilin–GFP mutant I, mutant K or K496R. Representative images for WT coilin–GFP and coilin–GFP mutant L with SMN probing to verify foci as CBs are shown in Fig. 6C. Collectively, these and other data examining the coilin–Nopp140 association (Courchaine et al., 2022) demonstrate that SUMOylation is a major driver of CB formation and regulates both CB size and number. We speculate that the SUMOylation of coilin and Nopp140 can influence interactions mediated by SIMs present on both proteins, resulting in a mechanism that governs the amount of coilin self-interaction and condensation, thereby controlling CB size and number (Fig. 6D). However, the involvement of SIMs in these interactions will need to be further studied.

## DISCUSSION

The mechanisms by which some cell types, but not others, form CBs and regulate CB size and number are not clear. Although SUMOylation is a known determinant in the formation of another subnuclear domain, the PML body (Zhong et al., 2000; Van Damme et al., 2010), the role that this PTM plays in CB formation is largely unexplored. Coilin, the CB marker protein, is known to be SUMOylated (Xiao et al., 2015; Lett et al., 2023), and the SUMO E3 ligase NSMCE2 contributes to coilin SUMOylation (Salas-Lloret et al., 2023). Reduction of NSMCE2 has been shown to increase CB number (Berchtold et al., 2018). Our results show that NSMCE2 KD decreases coilin SUMOylation and increases CB number (Fig. 1), in agreement with previous reports (Salas-Lloret et al., 2023; Berchtold et al., 2018). Inhibition of global SUMOylation using the SUMO activating enzyme (E1) inhibitor TAK-981 likewise reduced coilin SUMOylation and increased CB number (Fig. 2). Increased CB number was also observed in cells expressing coilin mutants in which lysines known or predicted to be SUMOylated were converted to arginine, such as coilin–GFP mutant L (Figs 3, 4). Collectively, the findings presented in Figs 1–4 support the idea that coilin SUMOylation is a regulator of CB number. Specifically, the data show that decreased SUMOylation increases CB number in HeLa cells and indicate that SUMOylation might in fact govern the number and size of CBs. In addition to SUMOylation, phosphorylation is another PTM that has been shown to regulate CB formation (Carrero et al., 2011; Hearst et al., 2009; Hebert and Matera, 2000). We hypothesize that these two PTMs work synergistically to regulate CB size and number, but more experimentation will be needed to validate this. Additionally, since coilin is known to be ubiquitylated on K496, it is likely that the balance of SUMOylation, phosphorylation and ubiquitylation contributes to coilin interactions and activities.

To explore the impact of SUMOylation on coilin, we first examined whether coilin interactions with Nopp140 are altered upon the mutation of known or predicted sites of coilin SUMOylation. Nopp140, which localizes to the nucleolus and CBs, plays an essential role in the biomolecular condensation of CBs by interacting with the coilin NTD and facilitating self-association (Courchaine et al., 2022; Meier and Blobel, 1990; Isaac et al., 1998). Using coimmunoprecipitation, we show that coilin–GFP mutant L recovers relatively more Nopp140 than coilin–GFP mutant I (Fig. 5). The mutant L and mutant I constructs both have the same 11 lysines converted to arginine, but the mutant L construct has one additional mutation, K84R. K84 resides in the region of coilin known to associate with Nopp140 and is strongly predicted to be SUMOylated. Our data suggest that the SUMOylation of K84 is a negative regulator of the coilin–Nopp140 interaction. This is particularly interesting due to the fact that mutation of a residue near to K84, D79, to an alanine has been found to decrease coilin–Nopp140 interaction (Courchaine et al., 2022), which is in direct contrast to what we saw when K84 was mutated to an arginine. Intriguingly these two residues are located between two coilin SIMs, reinforcing the idea that SUMOylation of coilin and Nopp140 is a major driver of CB formation.

To examine the CB formation potential of the coilin SUMO mutants more fully, we conducted studies in a primary cell line (HFFs) that has few CBs, as opposed to HeLa cells, which have many CBs. This analysis shows that the mutation of known or predicted sites of coilin SUMOylation increases CB number but decreases CB size (Fig. 6). Taken together, the findings presented here support the hypothesis that SUMOylation is a regulator of coilin–Nopp140 interaction and provide a mechanism for the cell to control both CB number and size. Since coilin and Nopp140 are both SUMOylated and contain SIMs, we propose a model wherein the interactions between coilin and Nopp140 are regulated by SUMOylation (Fig. 6D). Specifically, coilin–coilin interactions and coilin–Nopp140 interactions, along with interactions via the intrinsically disordered region of coilin (with nine known SUMOylated lysines), are influenced by SUMOylation. Our data further suggest that decreased coilin SUMOylation increases interaction with Nopp140, which results in less coilin–coilin interaction via the self-interaction domain. This model explains why we see an increased number of smaller CBs in the presence of coilin SUMO mutants. Since a GFP tag on the N terminus of coilin (GFP–coilin) decreases SUMOylation (Fig. S2D) (Lett et al., 2023) and results in a more nucleoplasmic phenotype compared to that seen with coilin–GFP (Fig. S3B), we suspect that the N-terminal GFP tag promotes coilin–Nopp140 interaction at the expense of coilin–coilin self-association. If this model is correct, cell lines with CBs should have a greater percentage of SUMOylated coilin compared to cell lines lacking CBs. Additionally, given that CBs play a role in small nucleolar RNP (snoRNP) biogenesis and several snoRNP component proteins are SUMOylated (Ryu et al., 2021), coilin might be part of the snoRNP group protein SUMOylation network that facilitates the interaction and stabilization of multicomponent protein complexes like snoRNPs.

## MATERIALS AND METHODS

### Cell lines, plasmids and transfections

The HeLa (transformed) cell line and human foreskin fibroblast (primary) cell line were obtained from the American Type Culture Collection (ATCC) and are free from contamination. Cells were cultured as previously described (Enwerem et al., 2014). All siRNAs are from Integrated DNA Technologies (Coralville, IA, USA) and used with RNAiMax (Invitrogen, Carlsbad, CA, USA) according to manufacturer's protocol. The negative control was previously described (Logan et al., 2018; Poole and Hebert, 2016). The sequences of the NSMCE2 A siRNA used are: forward, 5′-GAAGAUAUAAUUGUGACCCAAAGTC-3′; reverse, 5′-GAC UUUGGGUCACAAUUAUAUCUUCAU-3′. The sequences of the NSMCE2 B siRNA used are: forward, 5′-CUACAUUGGAUCGGCAACUAAACCA-3′; reverse, 5′-UGGUUUAGUUGCCGAUCCAAUGUAGCA-3′. All siRNA transfection was done for 72 h with the exception of NSMCE2 KD with His–SUMO-1 DNA transfection, which was a 96 h KD. All DNA transfections were 24 h with the exception of His–SUMO-1 transfection analysis of endogenous SUMOylated coilin, which used 48 h transfection. The GFP–coilin (Hebert and Matera, 2000) and coilin–GFP plasmids (Shpargel et al., 2003) previously contained a point mutation of K496E that was shown to impact CB formation (Basello et al., 2022). For the experiments shown here, we modified these plasmids to use WT sequences with K496 unless a mutant is specified. WT myc–coilin was obtained from the Lamond lab (University of Dundee, UK). GFP empty vector is pEGFP-C3 C-terminal fusion protein fusion vector, GenBank Accession #U57607. The His-SUMO-1 construct was ordered from Addgene (#17271, deposited by the laboratory of S. Goff).

### Mutagenesis

Mutants used in this study were constructed using Q5 site-directed mutagenesis kit (New England Biolabs, Ipswich, MA, USA). All lysine residues mutated were changed to arginine residues generated by point mutations. All constructs were verified by sequencing (Eurofins Genomics, Louisville, KY, USA).

### Drug treatment

TAK-981 (Thermo Fisher Scientific, Waltham, MA, USA) treatment was done for 24 h at a concentration of 0.1 µM. DMSO was used as the vehicle control. The same amount of DMSO that was used to reconstitute TAK-981 was used during control treatment.

### Western blotting

Cells were lysed using RIPA containing Protease Inhibitor Cocktail (PIC; Thermo Fisher Scientific) as previously described (Poole and Hebert, 2016). Lysate was run on a 10% Mini-Protean Gel (Bio-Rad Laboratories, Hercules, CA, USA). Western transfer was done via a Trans-Blot Turbo Transfer System (Bio-Rad Laboratories). Detection was conducted as previously described (Poole and Hebert, 2016). The primary antibodies used were anti-β-actin mouse monoclonal antibody (1:5000; 8H10D10; Cell Signaling Technology, Danvers, MA, USA); anti-NSMCE2 polyclonal antibody (1:1000; 13627-1-AP; Proteintech, Rosemont, IL, USA), anti-GFP monoclonal antibody (1:500; 11814460001; Roche, Germany), anti-SUMO-1 polyclonal antibody (1:1000; 10329-1-AP; Cell Signaling Technology, Danvers, MA, USA), anti-SUMO-2/3 polyclonal antibody (1:1000; 11251-1-AP; Cell Signaling Technology, Danvers, MA, USA), anti-coilin polyclonal antibody (1:1000; sc-32860; Santa Cruz Biotechnology, Dallas, TX, USA), anti-NOCL1 (Nopp140) polyclonal antibody (1:1000; 11815-1-AP; Proteintech) and anti-myc (1:1000; sc-40; Santa Cruz Biotechnology). Secondary antibodies used were HRP-conjugated goat anti-mouse-IgG (1:5000; PIA32742; Thermo Fisher Scientific) and HRP-conjugated goat anti-rabbit-IgG (1:5000; Pi31460; Thermo Fisher Scientific). Bands were detected using SuperSignal West Pico Chemiluminescent Substrate (Thermo Fisher Scientific) following the manufacturer's protocol. Imaging was done using a ChemiDoc (Bio-Rad Laboratories). Bands were quantified using Image Lab Software 6.0.1 (Bio-Rad Laboratories). Any adjustments were done to the entire blot. GraphPad Prism was used for statistical analysis, using a two-tailed unpaired Student's *t*-test for bar chart generation. Full western blots for each figure are shown in Fig. S4.

### Ni-NTA pulldown assays

Cells were lysed in RIPA minus EDTA (1 M Tris-HCl pH 7.6, 5 M NaCl, 1% NP-40, 0.1% SDS and 1% sodium deoxycholate) with PIC also minus EDTA (Thermo Fisher Scientific). Included in the RIPA+PIC buffer was 20 µl/ml of 1 M *N*-ethylmaleimide to prevent deSUMOylation. Collected lysate was incubated with 50 µl of 50% Ni-NTA beads (QIAGEN, Germantown MD USA) for 1 h at 4°C. Following incubation, the beads were washed three times with RIPA minus EDTA buffer. Then, 20 µl of 2× SDS loading buffer (2 ml 100% glycerol, 4 ml 10% SDS, 2 ml 500 mM Tris-HCl pH 6.8, 2 ml 1M DTT and 0.25 mg Bromophenol Blue divided into 1 ml aliquots) was added to the beads, and samples were ready for visualization via western blotting.

### Immunofluorescence

HeLa and HFF cells were seeded on 2- or 4-well CC2 slides (Thermo Fisher Scientific). Cells were fixed in 4% paraformaldehyde followed by permeabilization in 0.5% Triton X-100 and blocking in 10% normal goat serum (NGS; Thermo Fisher Scientific), as previously described (Logan et al., 2018). CBs were detected with 1:200 anti-coilin polyclonal antibody (H-300; Santa Cruz Biotechnology) or 1:200 anti-coilin monoclonal antibody (F7, sc-55594; Santa Cruz Biotechnology). Nopp140 was detected with 1:50 anti-NOCL1 (Nopp140) polyclonal antibody (11815-1-AP; Proteintech). SMN was detected with 1:250 anti-SMN (2B1) monoclonal antibody (Novus, Centennial, CO, USA). Antibody incubations were performed in the presence of 10% NGS at 37°C for 30 min for both primary and secondary antibodies. Secondary antibodies used were goat anti-mouse-IgG Alexa Fluor 594 and goat anti-rabbit-IgG Alexa Fluor Plus 488 (both Invitrogen at 1:200). Slides were then washed in PBS and stained with DAPI to detect nuclei. Coverslip mounting was done with Antifade (Invitrogen). Cells were imaged as previously described (Logan et al., 2020). For statistical analysis of HFF CB number and size and HeLa CB count, a two-tailed unpaired Student's *t*-test was used.

### Immunoprecipitation

HeLa cells were lysed and collected in RIPA buffer plus PIC (Thermo Fisher Scientific). Lysates were sonicated three times with a Fisher Scientific sonicator (model 100) for 5 s each using the output setting of 1. Lysates were then centrifuged at 113 ***g*** 12,000 rpm for 15 min at 4°C. Lysates were subject to immunoprecipitation using 50 µl of 50% protein G beads (Thermo Fisher Scientific) and 2 µg anti-GFP mouse monoclonal antibody (11814460001; Roche, Indianapolis, IN, USA). Immunoprecipitates were washed three times with RIPA buffer followed by addition of 20 µl of 2× SDS loading buffer, after which samples were ready for visualization via western blotting.

### Flow cytometry

Flow cytometry analysis was performed on HeLa cells in the following groups: untreated for morphology and cell cycle distribution normalcy assessment, control siRNA, NSMCE2 A siRNA, DMSO and TAK-981 (0.1 μM). Cells were seeded into 6-well plates. Treatment and transfection conditions were identical to those corresponding protocols done for the Ni-NTA pulldown for each condition. Cells were harvested for flow cytometry analysis with ∼1,000,000 cells/sample. Samples were stained using FxCycle PI/RNase Staining Solution (Invitrogen) for 30 min and run on a Novocyte flow cytometer (ACEA). The Dean–Jett–Fox model [Novocyte flow cytometer (ACEA)] was used to show the distribution of cell cycles in samples. Data were analyzed in GraphPad Prism to determine significance by two-tailed unpaired Student's *t*-test.

### SuperPlot construction

All SuperPlots were constructed using https://huygens.science.uva.nl/SuperPlotsOfData/. Formatting data for uploading was done via Excel per instructed organization (Goedhart, 2021). All data was converted to ‘tidy’. Data display was set to ‘Data & Distribution’ for all plots. All statistical analysis was done in GraphPad Prism and significance was added after SuperPlot construction. Analysis was done using two-tailed unpaired Student's *t*-tests.

## Supplementary Material

10.1242/joces.263447_sup1 Supplementary information
