# Supplementary material for: Cajal body formation is regulated by coilin SUMOylation
Source: J Cell Sci. 2024 Dec 11;137(23):jcs263447. doi: 10.1242/jcs.263447 (PMC11827600; doi:10.1242/jcs.263447)
Supplement: Supplementary information [file joces-137-263447-s1.pdf]

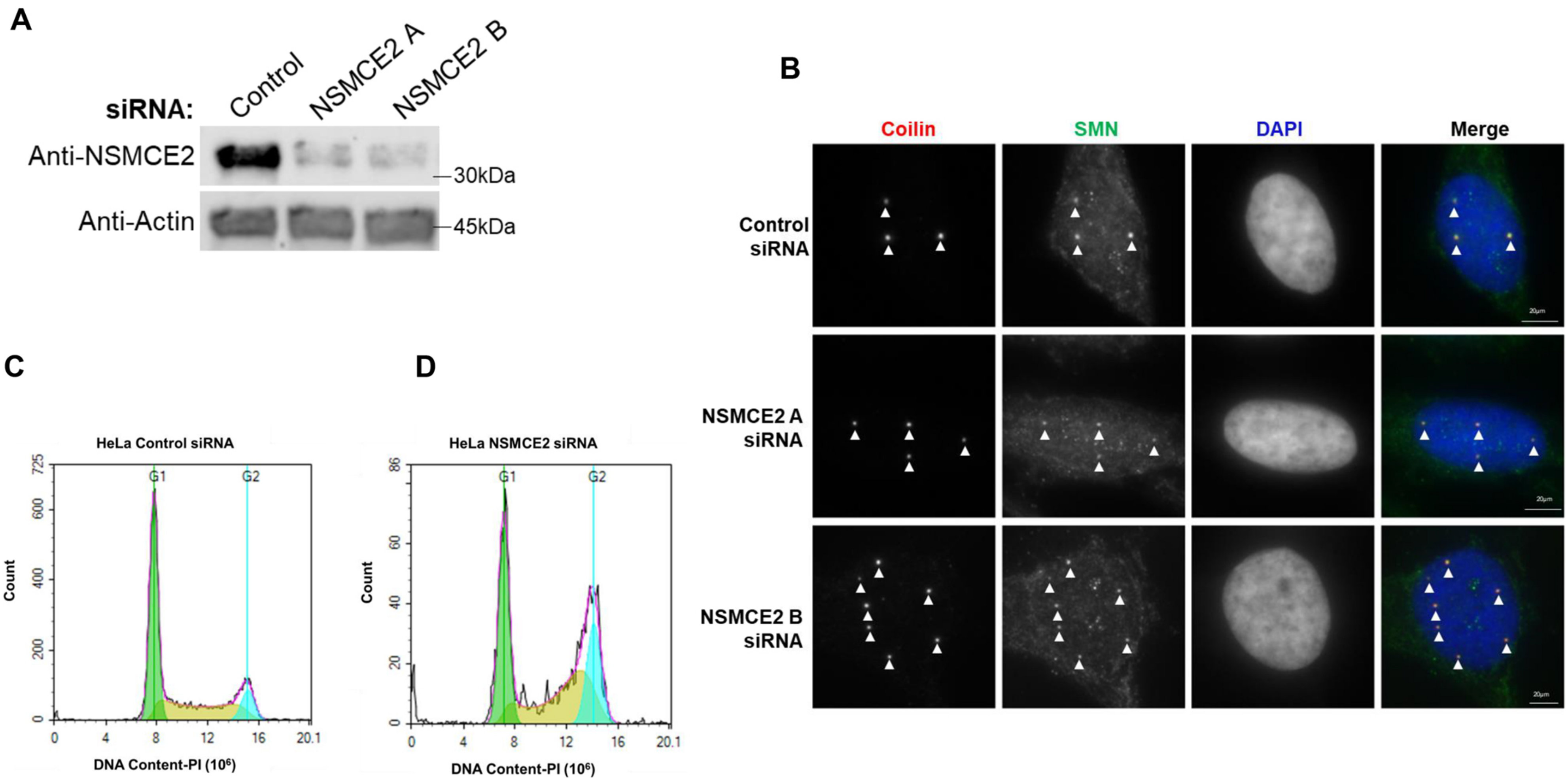

Fig. S1. A) HeLa cells were knocked down with negative control siRNA or two different types of NSMCE2 siRNAs for 72 h. Lysate was subjected to SDS-PAGE, Western blotting, and probing for NSMCE2 and beta-actin. B) Immunofluorescence of HeLa cells transfected with control siRNA, NSMCE2 A siRNA, or NSMCE2 B siRNA for 72 h. Coilin signal is shown in red. SMN staining is in green. Arrow heads are used to denote some CBs. Scale bars for each image show relative size. C) HeLa cells transfected with control siRNA for 72 h and examined with flow cytometry to assess cell cycle phase distribution. ~1,000,000 cells. D) HeLa cells transfected with NSMCE2 siRNA for 72 h and examined with flow cytometry to assess cell cycle phase distribution. ~1,000,000 cells.

Journal of Cell Science • Supplementary information

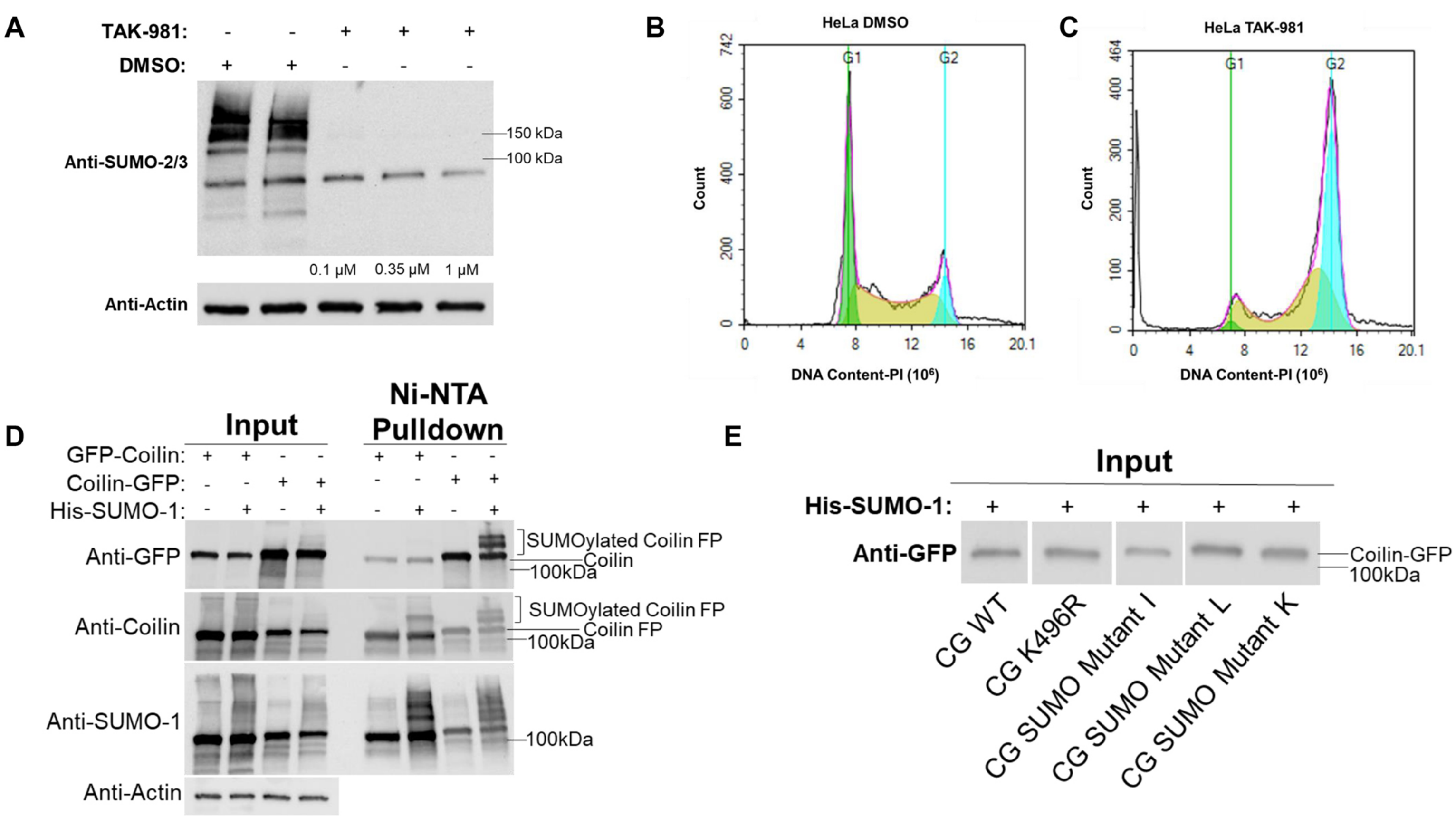

Fig. S2. A) HeLa cells were treated with different concentrations of TAK-981 or DMSO for 24 h. Lysate was subjected to SDS-PAGE, Western blotting and probing for SUMO- 2/3 and beta-actin. B) HeLa cells treated with DMSO for 24 h and examined with flow cytometry to assess cell cycle phase distribution. ~1,000,000 cells. C) HeLa cells treated with TAK-981 for 24 h and examined with flow cytometry to assess cell cycle phase distribution. ~1,000,000 cells. D) HeLa cells were transfected with GFP-coilin or coilin-GFP with or without His-SUMO-1 for 24 h. Input and Ni-NTA pulldown reactions were subjected to SDS-PAGE, Western blotting and probing for GFP, coilin, SUMO-1, and beta-actin. E) Inputs of coilin-GFP WT and mutants.

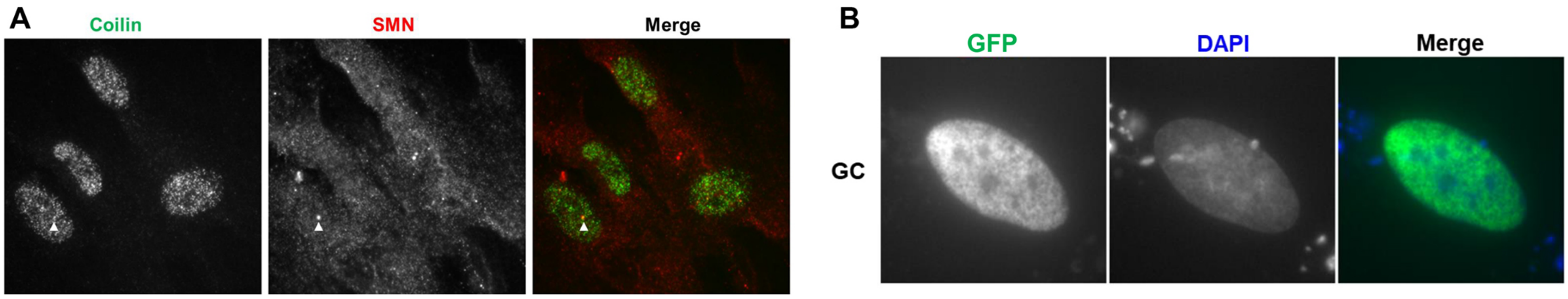

Fig. S3. A) Immunofluorescence of HFF cells stained for coilin and SMN. A CB containing coilin and SMN is indicated by an arrowhead in one of the cells. B) Immunofluorescence of HFF cells transfected with GFP-coilin (GC). GFP-coilin is shown in green.

Journal of Cell Science • Supplementary information

Figure 1A:

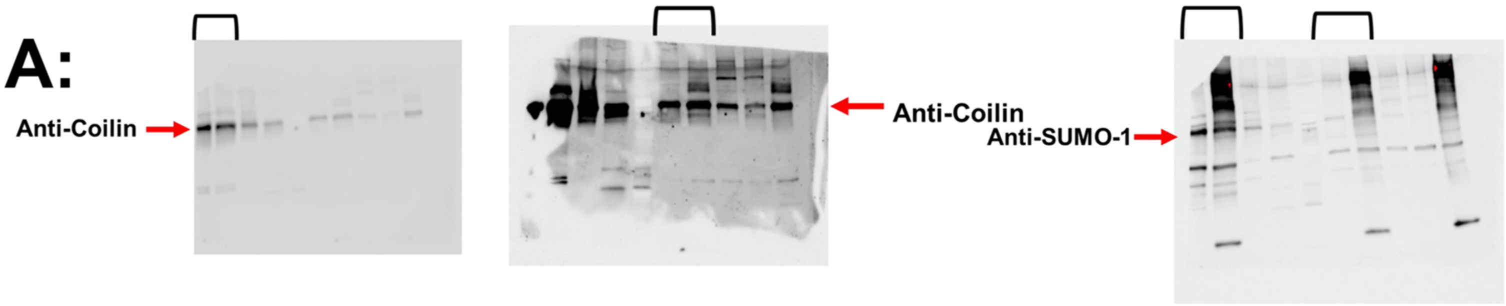

Figure 1B:

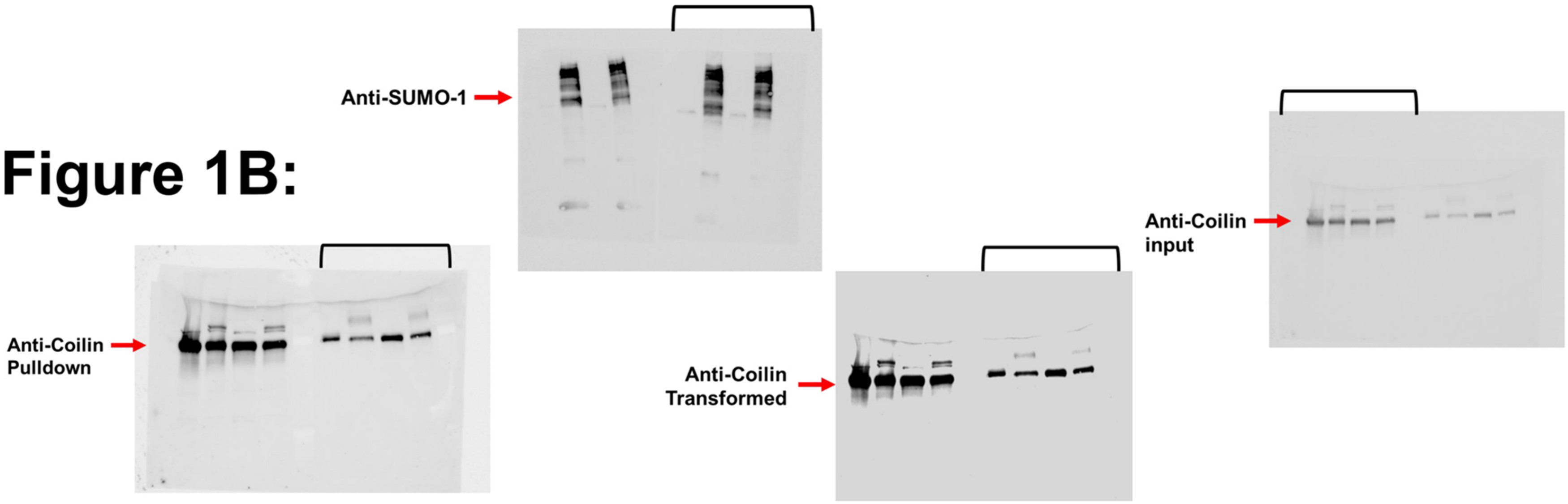

Figure 2A:

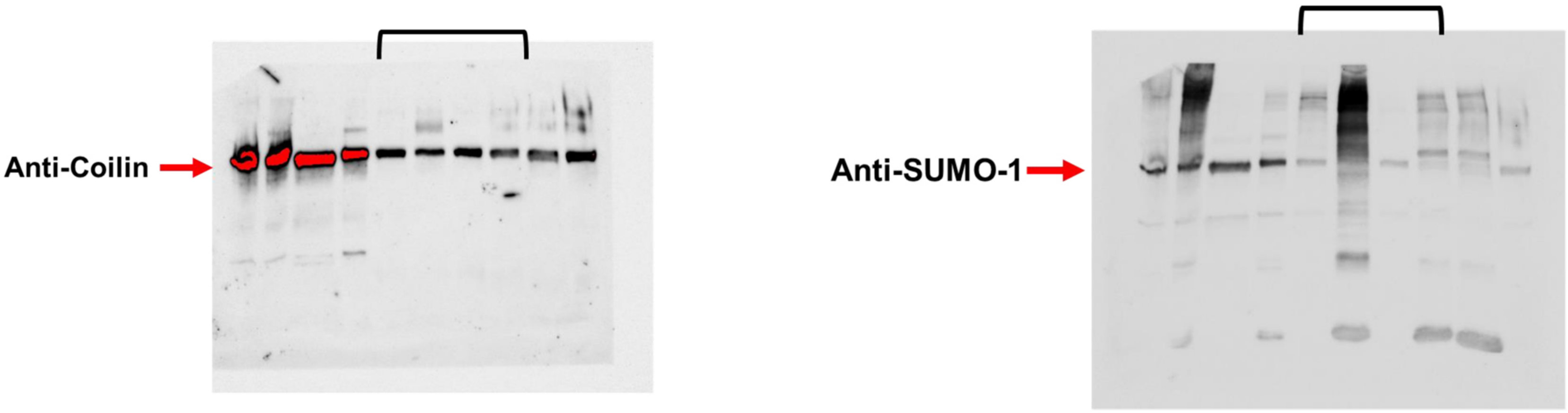

Journal of Cell Science • Supplementary information

Figure 3B:

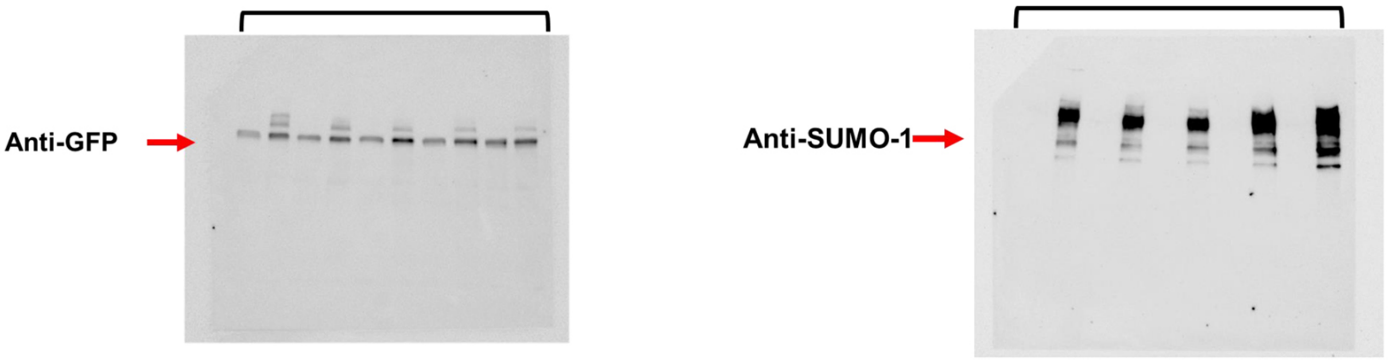

Figure 3D:

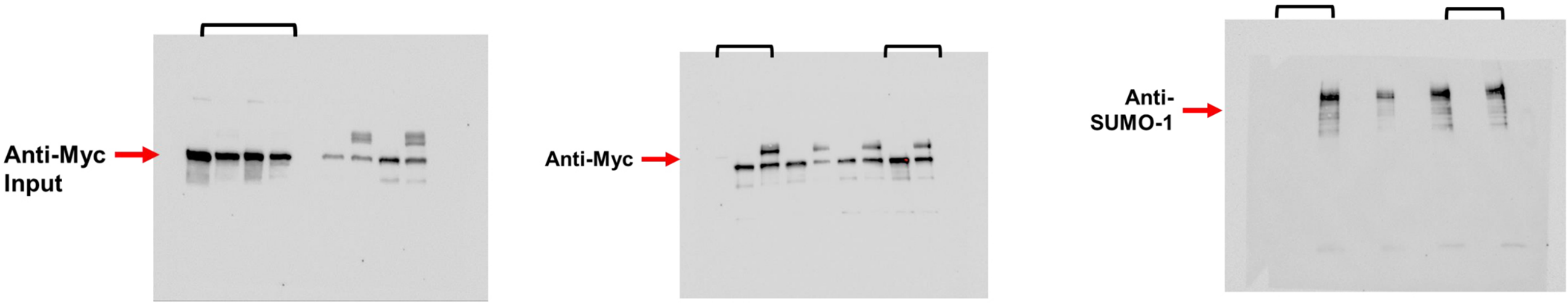

Figure 5A:

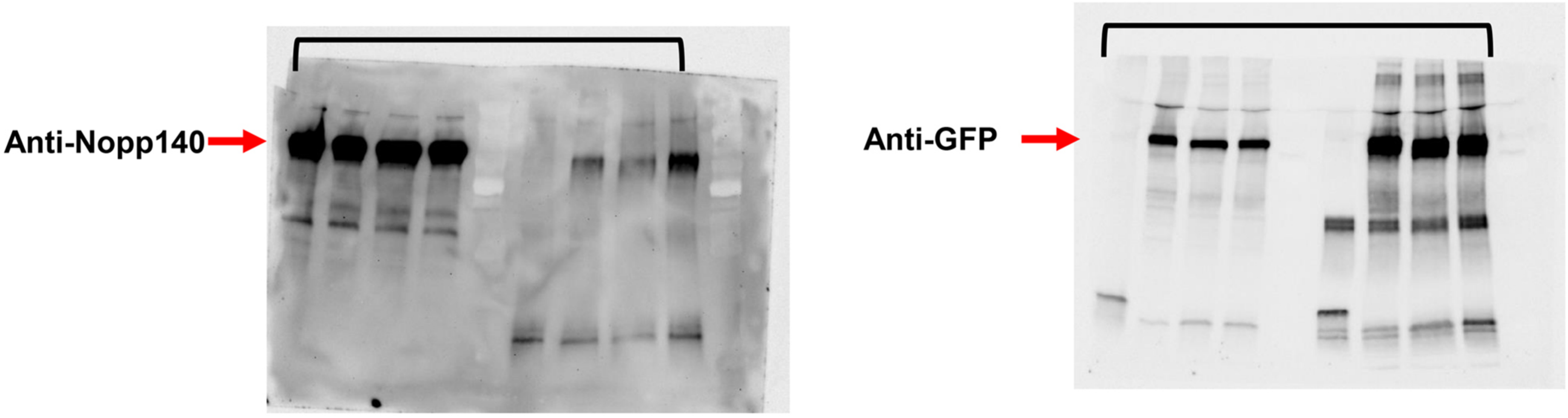

Journal of Cell Science • Supplementary information

Supplemental Figure 1A:

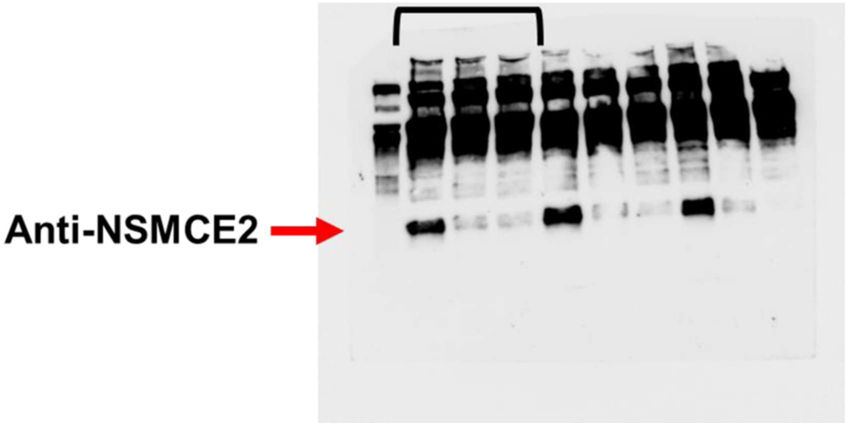

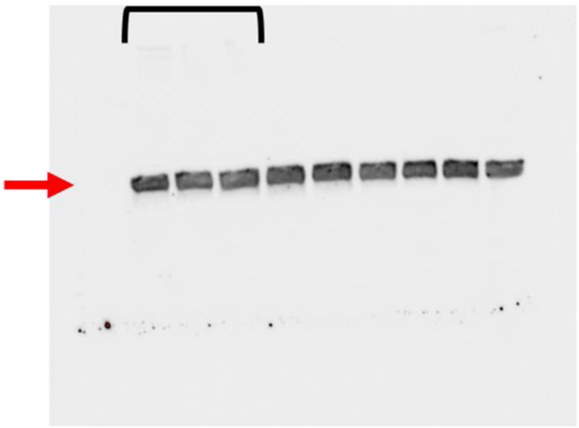

Supplemental Figure 2A:

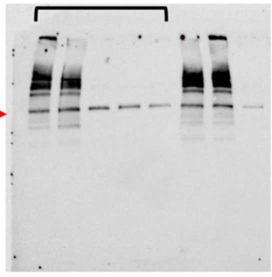

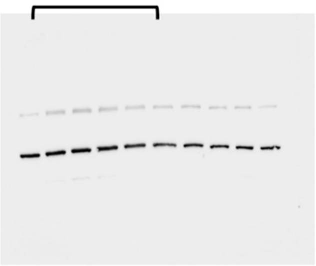

Supplemental Figure 2D:

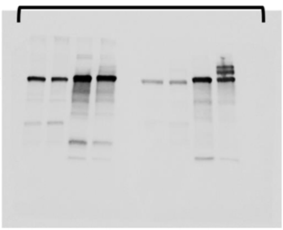

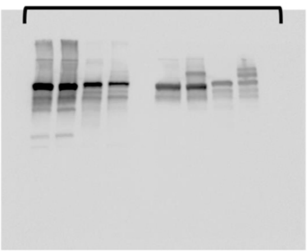

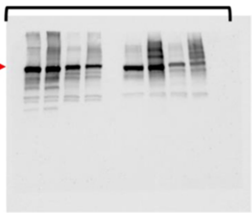

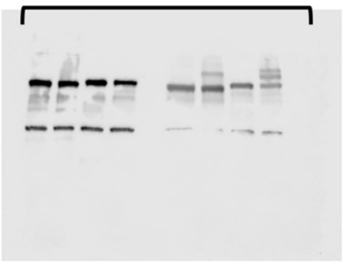

Supplemental Figure 2E:

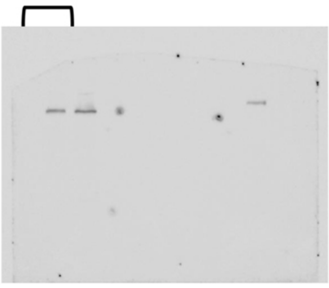

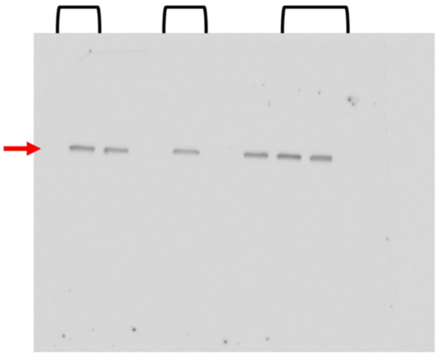

Fig. S4. Full Western blots used to generate figures.

Journal of Cell Science • Supplementary information
